# The prognostic value of tumor mutation burden (TMB) and its relationship with immune infiltration in breast cancer patients

**DOI:** 10.1186/s40001-023-01058-x

**Published:** 2023-02-20

**Authors:** Shengjin Cui, Jingying Feng, Xi Tang, Shuang Lou, Weiquan Guo, Xiaowei Xiao, Shuping Li, Xue Chen, Yu Huan, Yiwen Zhou, Lijia Xiao

**Affiliations:** Department of Clinical Laboratory, Shenzhen Hospital, Southern Medical University, No. 1333 of Xinhu Road, Shenzhen, 518101 Guangdong China

**Keywords:** Breast cancer, Tumor mutation burden (TMB), Immune infiltrate, Survival

## Abstract

**Objective:**

Although the tumor mutation burden (TMB) was reported as a biomarker for immunotherapy of various cancers, whether it can effectively predict the survival prognosis in breast cancer patients remains unclear. In this study, the prognostic value of TMB and its correlation with immune infiltration were explored by using multigroup studies.

**Methods:**

The somatic mutation data of 986 breast cancer patients were obtained from TCGA database. Breast cancer patients were divided into a low-TMB group and a high-TMB group according to the quartile of TMB scores. The differentially expressed genes (DEGs) were identified by the “limma” R program. The CIBERSORT algorithm was utilized to estimate the immune cell fraction of each sample. The TIMER database was utilized to evaluate the association between CNVs of immune genes and tumor immune cell infiltration and the prognostic value of the immune cells in breast cancer.

**Results:**

In breast cancer, *TP53*, *PIK3CA*, *TTN*, *CDH1* and other genes were the most important mutated genes. Higher survival rate of patients was found in the low-TMB group. Among the top 10 DEGs, three of them belong to the KRT gene family. GSEA enrichment analysis showed that MAPK, Hedgehog, mTOR, TGF-bate and GnRH signaling pathways were enriched in the low-TMB group. The infiltration levels of the most of immune cells were higher in the low-TMB group (*P* < *0.01*). Higher expression of *CCL18* and *TRGC1* was correlated with poor prognosis. Breast cancer patients with *CCL18* copy number variations, especially arm-level gains, showed significantly decreased immune cell infiltration. In the low B cell infiltration group, the survival prognosis of breast cancer patients was poor.

**Conclusions:**

TMB is a potential prognosis marker in breast cancer. Immune-related gene *CCL18* and *TRGC1* are biomarkers of poor prognosis while immune (B cell) infiltration is a biomarker of good prognosis.

## Introduction

Breast cancer is the most common gynecological cancer worldwide, and it ranks second in female cancer deaths [1]. The incidence rate of breast cancer has still increased year by year in China (27.24 million cases in 2015 but 367,900 cases in 2018) [2, 3]. Although improvements in early detection and treatment have reduced breast cancer mortality by 38%, metastasis of breast cancer at is the major reason of its high lethality [4–6]. These reports demonstrate that development of innovative treatments for reducing recurrence and death in breast cancer are urgently needed. In recent years, immune checkpoint blockade (ICB) therapy has successfully improved overall survival (OS) in a variety of human cancers, including renal cell carcinoma [7], advanced melanoma [8], non-small-cell lung cancer [9] and bladder urothelial carcinoma [10]. The success of ICB therapy has rekindled the hope for immune-based therapy in breast cancer. Increasing data suggested that the immune system plays a decisive role in breast cancer patients’ response to standard therapy and the long-term survival rate [11].

Tumor mutation burden (TMB), which was reported to be used to predict the efficacy of ICB, is associated with a high neoantigen burden, high T cell infiltration and a high response rate to immune checkpoint inhibitors (ICIs) in different tumor types. TMB has emerged as a useful biomarker for evaluation of immunotherapy effectiveness in several cancer types, but its value is not well understood in breast cancer [12–17]. Herein, this study aimed to investigate the prognostic value of TMB and its relationship with immune cell infiltration in breast cancer.

## Materials and methods

### Clinical data source and processing

The simple nucleotide variation data of the breast cancer dataset and RNA-seq data (HTSeq-FPKM data) of breast cancer samples and normal tissue samples were downloaded from TCGA database (https://cancergenome.nih.gov/). The mutation annotation format (MAF) file was analyzed and visualized using the “maftools” R software package.

### Calculation of TMB and survival analysis

TMB represents the total amount of somatic mutations per megabase (Mut/Mb) of DNA, including deletions, insertions, substitutions, and translocations. In this research, the mutation frequency was calculated by running a Perl script based on strawberry-perl-5.30.0.1–64 bits. The quartile of the TMB score was used as the threshold to divide breast cancer samples into low-TMB group and the high-TMB group. The “survival” R package was utilized to evaluate the effect of TMB on the survival prognosis in breast cancer patients. In addition, the correlation between TMB levels and clinical pathological characteristics was analyzed by using the "cliCor." R package.

### Screening and functional analysis of DEGs

The “limma” R package was used to identify the differentially expressed genes (DEGs) between the two groups, and the criteria were set as follows: *Fold Change (FC)* = *2* and *False Discovery Rate (FDR)* < *0.05*. The “GO” and “KEGG” R software packages were applied to perform Gene Ontology (GO) analysis and Kyoto Encyclopedia of Genes and Genomes (KEGG) analysis, respectively. The abundance bar charts and point charts were plotted by using “DOSE”, “enrichplot” and “ggplot2” R software packages.

GSEA 4.1.0 was utilized to perform gene set enrichment analysis (GSEA) in terms of the TMB level as the phenotype. The internal reference gene set was “c2.cp.kegg.v7.0.symbols.gmt” that obtained from the GSEA-MSigDB database (http://software.broadinstitute.org/gsea/msigdb/). *FDR* < *0.25* indicated that the gene sets were significantly enriched. A gene list containing 2347 immune-related genes were obtained from the immunology database and analysis portal (Immport) (https://www.immport.org/home). The differentially expressed immune genes between two groups were selected by the “Venn Diagram” R package.

### CIBERSORT algorithm analysis and TIMER database analysis

The immune cell fraction of each sample was estimated by the CIBERSORT algorithm (R script v1.03). After using the “limma” R package to standardize the transcription data, samples with *P* > *0.05* were excluded and the rest of 1009 samples were used for further analysis. By running the “barplot. R” package, the relative level of immune cells in each sample was displayed in the form of a histogram. The Wilcoxon rank sum test was used to analyze the difference in the abundances of 22 immune cell infiltrations between the low-TMB group and the high-TMB group. The violin map was drawn by using the “vioplot. R” package.

With *| FC |*> *2* and *FDR* < *0.05* as the threshold, we identified 62 immune-related differential genes and then analyzed their correlation with the survival rate of breast cancer patients. We used the “SCNA” module of the TIMER database to analyze the association between copy number variations (CNVs) of immune-related genes and immune cell infiltration in tumor tissue (https://cistrome.shinyapps.io/timer/). The "Survival" module of the TIMER database was used to evaluate the survival prognosis of immune-related genes and immune cells in breast cancer patients.

## Results

### Mutations in breast cancer samples

**Fig. 1:**
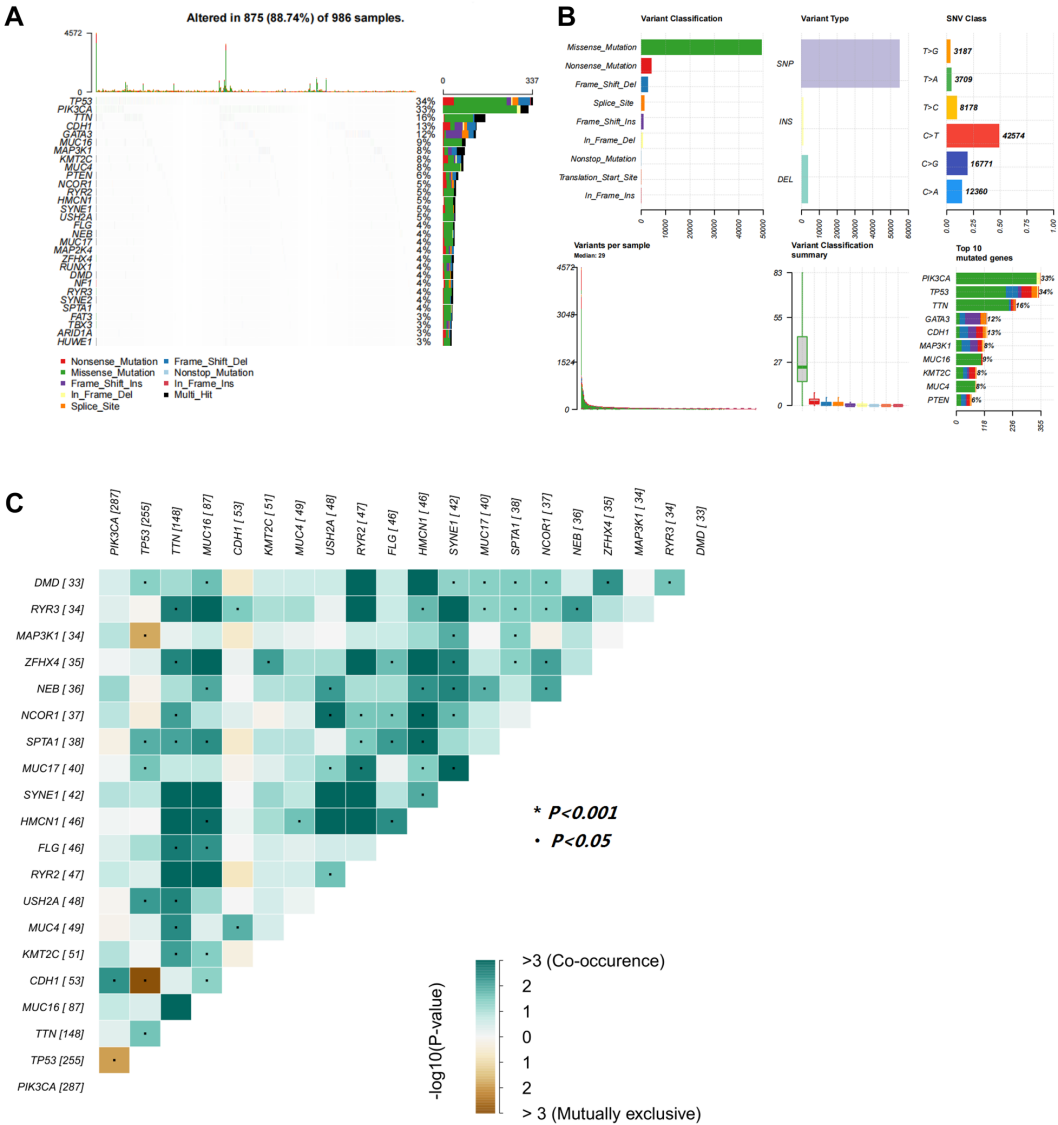
Mutation profiles in breast cancer samples. **A** Mutation landscape plot of breast cancer samples. **B** Variation classification, variation type, and SNV category in breast cancer samples. **C** Exclusivity and co-occurrence among mutated genes. *SNP* single nucleotide polymorphism; *SNV* single nucleotide variant

The mutation data of 994 breast cancer samples were obtained from TCGA database. A total of 986 valid data points were screened. The landscape of mutation profiles was visualized by using the “maftools” R package. It was shown that 88.74% (875) of patients carried somatic mutations. The top 10 mutated genes in breast cancer samples were *TP53* (34%), *PIK3CA* (33%), *TTN* (16%), *CDH1* (13%), *GATA3* (12%), *MUC16* (9%), *MAP3K1* (8%), *KMT2C* (8%), *MUC4* (8%) and *PTEN* (6%) (Fig. 1A). Missense mutations ranked first in the variation classification. Single nucleotide polymorphisms (SNPs) were the predominant variant type. The most common single nucleotide variant (SNV) was C > T conversion in breast cancer samples (Fig. 1B). The co-occurrence and exclusive analysis showed that gene expression of *TP53* and *CDH1* were mutually exclusive, while the co-occurrence was found between NCOR1 and HMCN1, NCOR1 and USH2A, HMCN1 and SPTA1, HMCN1 and MUC16 (Fig. 1C).

### Survival prognosis of TMB and its correlation with clinical pathological characteristics

**Fig. 2:**
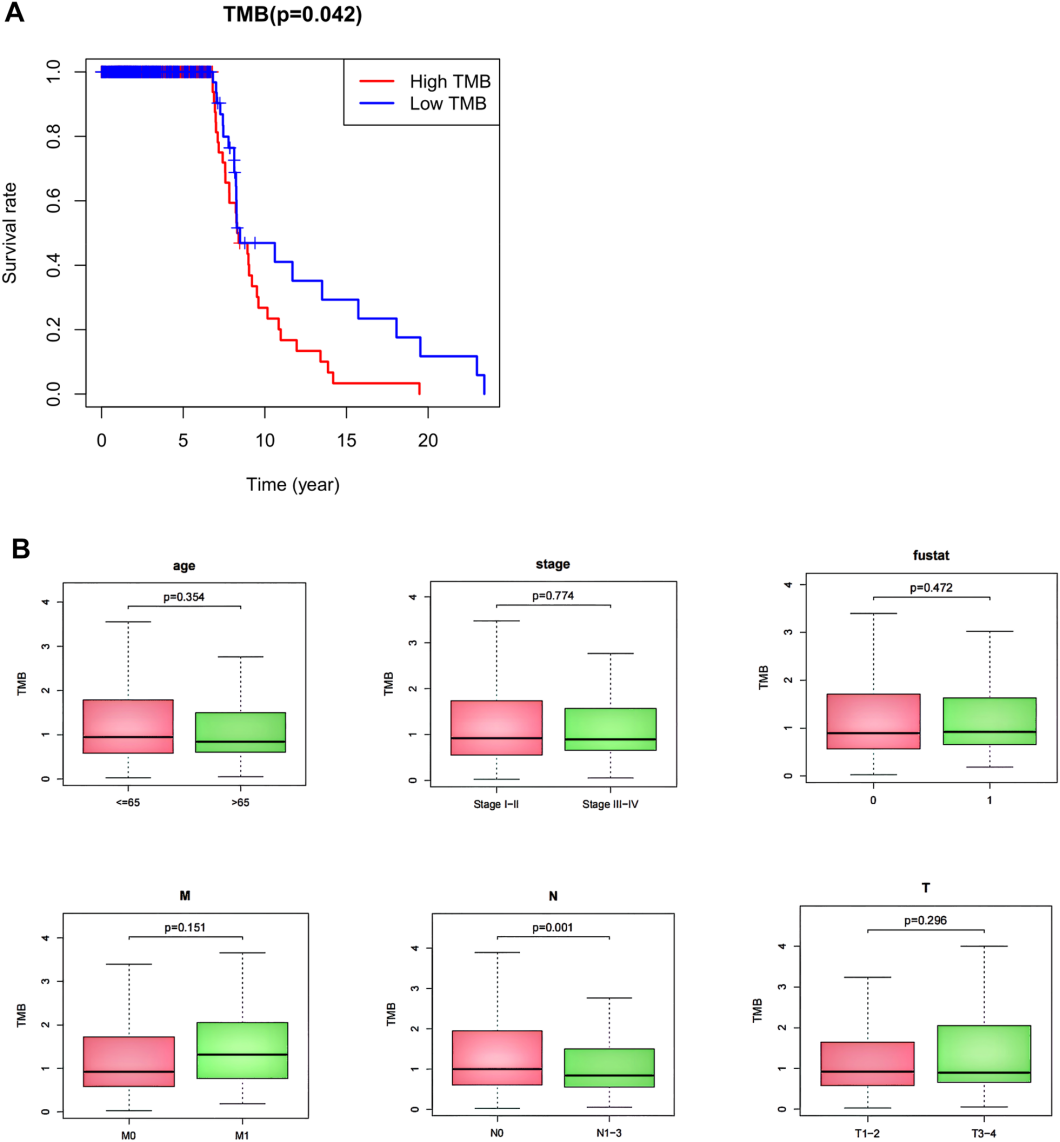
The prognostic value of TMB score and its correlation with clinical pathological characteristics. **A** The survival curves of the low- and high-TMB groups. **B** The correlation between TMB and clinical pathological characteristics. TMB, tumor mutational burden

The TMB score of 986 breast cancer patients was calculated. According to the quartile of the TMB score, the breast cancer patients were divided into the low-TMB group and the high-TMB group. The survival analysis showed that the low-TMB group had favorable survival outcome (Fig. 2A) (*P* = *0.048*). TMB scores were significantly associated with regional lymph node metastasis (N) (*P* < *0.05*), but not with other clinical pathological characteristics in breast cancer (*P* > *0.05*) (Fig. 2B).

### Comparison of gene expression profiles between the low- and high-TMB groups

**Fig. 3:**
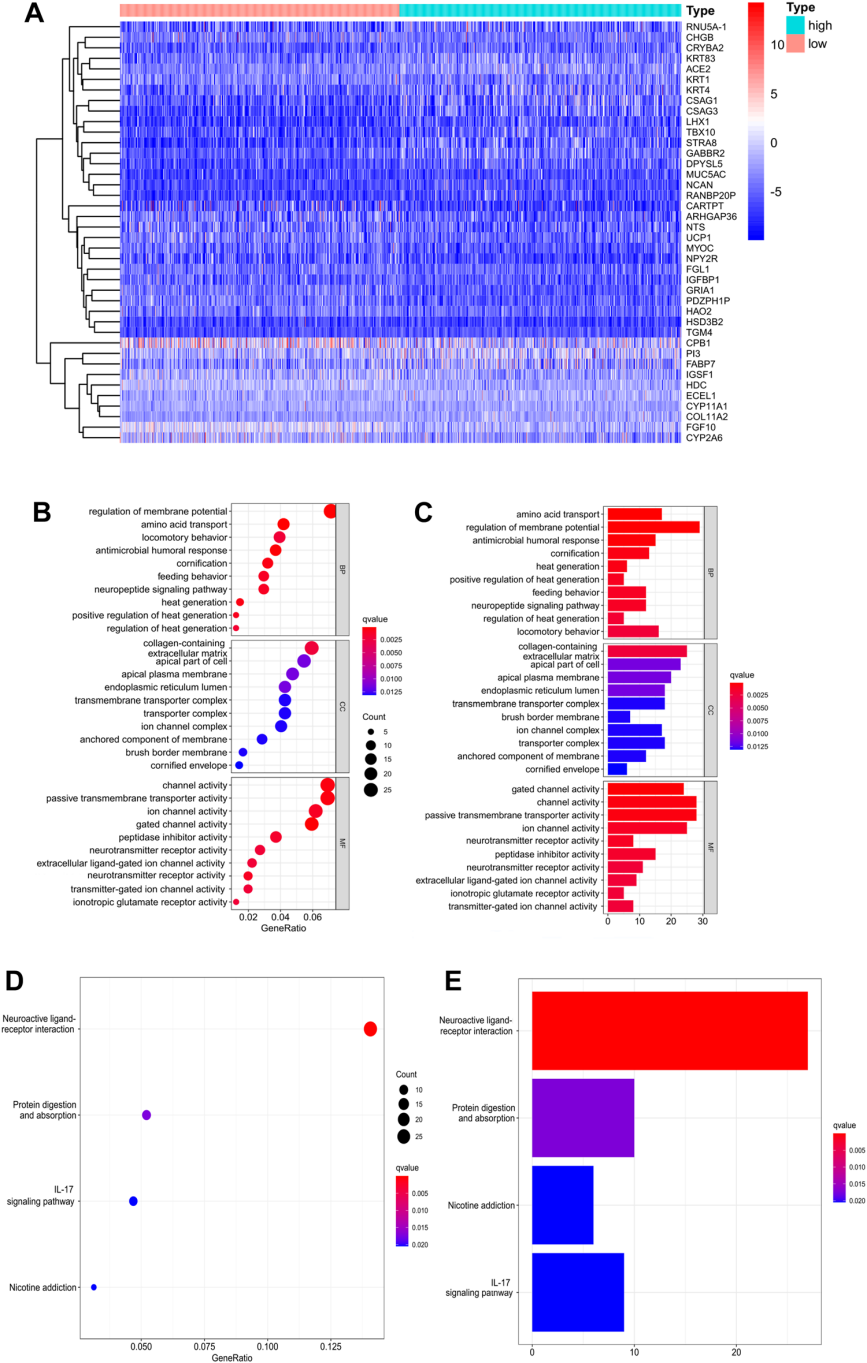

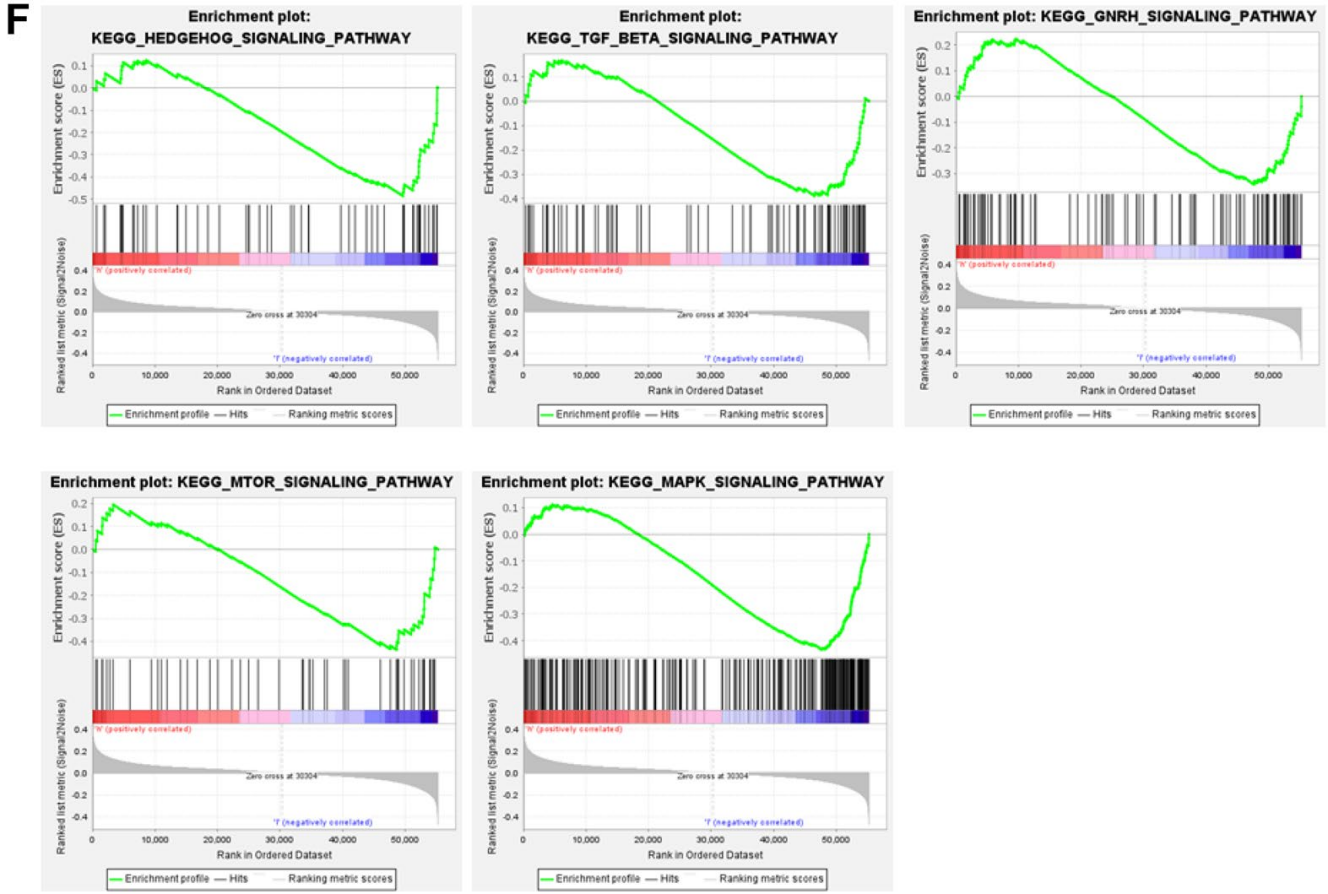
Comparison of gene expression profiles between the low-TMB and high-TMB groups. **A** The top 40 DEGs are shown in a heatmap plot. **B**, **C** The abundance bar charts and point charts of GO analysis. **D**, **E** The abundance bar charts and point charts showing the KEGG analysis results. **F** The GSEA results showed that immune-related [Hedgehog, TGF-b, GnRH mTOR, and MAPK] signaling pathways were enriched in the low-level TMB group. DEGs, differentially expressed genes; TMB, tumor mutation burden

A total of 598 DEGs were identified through differential expression analysis, among which 342 genes were upregulated and 256 genes were downregulated. The threshold was set as *log2FC* > 1 and *FDR* < *0.05*. The top 40 DEGs are displayed in a heatmap plot (Fig. 3A). In the top 10 DEGs, 3 of them (*KRT83*, *KRT4* and *KRT1*) belong to the KRT gene family. *KRT83* and *KRT1* were upregulated in the low-TMB group, while *KRT4* was downregulated. GO enrichment analysis showed that the DEGs were involved in many biological functions, such as regulation of membrane potential, collagen-containing extracellular matrix, channel activity, passive transmembrane transporter activity and others (Fig. 3B, C). DEGs were mainly enriched in neuroactive ligand-interaction receptors in the results of KEGG enrichment analysis (Fig. 3D, E). In addition, GSEA enrichment analysis showed that the MAPK, mTOR, GnRH, TGF-bate and Hedgehog signaling pathways were mainly enriched and downregulated in the low-TMB group (Fig. 3F).

### Immune cell infiltration and immune-related DEG analysis

**Fig. 4:**
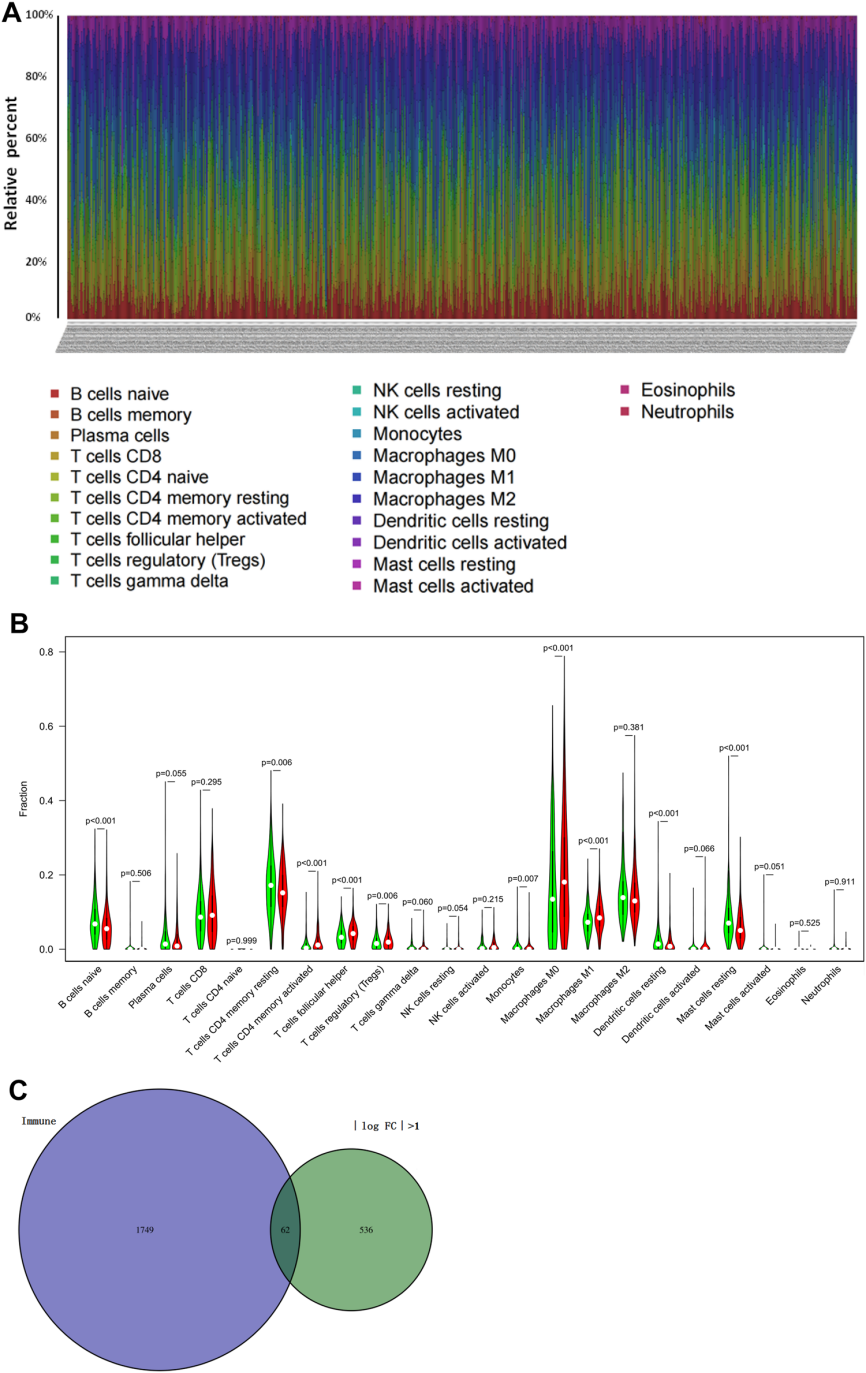
Immune cell infiltration in low- and high-TMB groups and immune-related DEGs analysis. **A** The relative percentage of 22 subtypes of immune cells in each sample was described by histogram plot. **B** The violin diagram shows the comparison of immune cell infiltration between the low-TMB and high-TMB groups. Green represents the low-TMB group, and red represents the high-TMB group. **C** Sixty-two DEGs associated with immunity were identified using a Venn diagram

The abundance of 22 immune cells in low- and high-TMB groups was evaluated using the CIBERSORT algorithm. A total of 1009 samples were screened after excluding samples with *P* > *0.05*. A histogram plot was used to show the relative percentage of 22 immune cells in each breast cancer sample (Fig. 4A). Naive B cells, memory CD4 T cells and resting mast cells were significantly infiltrated in the low-TMB group (*P* < *0.01*), while macrophages M0 and M1 were significantly infiltrated in the high-TMB group (*P* < *0.01*) (Fig. 4B). A total of 2498 immune-related genes were downloaded from Immport. A total of 62 genes of them were obtained by running the “Venn Diagram” R package, regarding as immune-related DEGs (Fig. 4C).

### Survival prognosis of immune-related genes

**Fig. 5:**
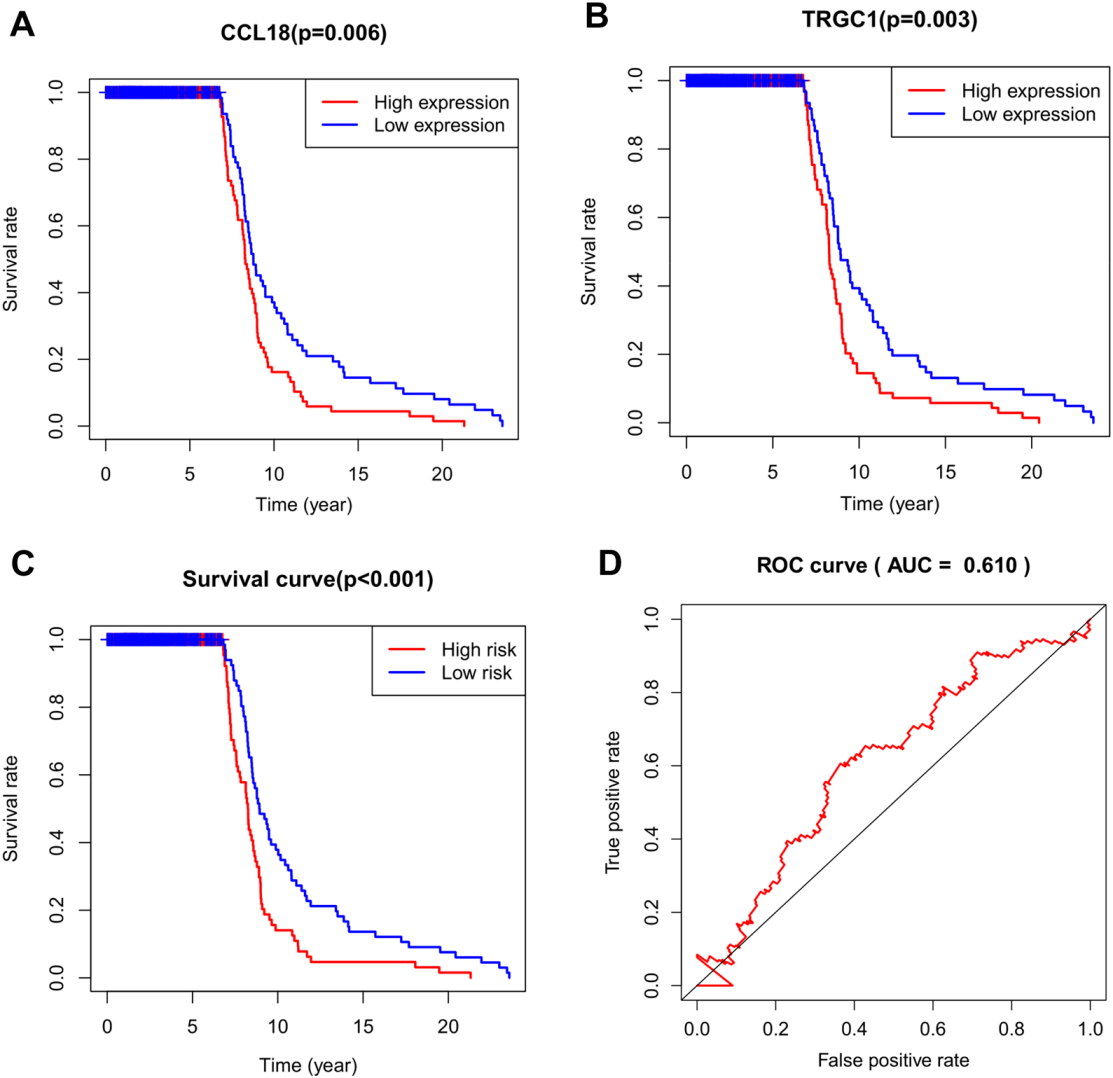
Survival prognosis of immune-related genes. **A**, **B** The survival curve of *CCL18* and *TRGC1* immune genes showed that the low expression group had a good prognosis. **C** The high-risk group was associated with poor survival. D The ROC curve confirmed the reliability of the risk survival curve (AUC = 0.610)

The survival analysis of 62 immune-related DEGs was conducted using multivariate Cox regression analysis on the “survival” module of the TIMER database. *CCL18* and *TRGC1* were highly correlated with survival outcome in breast cancer patients. Breast cancer patients with higher expression of *CCL18* and *TRGC1* had poorer survival (Fig. 5A, B). The risk score of the two genes was calculated, and the breast cancer patients were divided into low-risk and high-risk groups based on the median risk score. The survival curve showed that breast cancer patients in the high-risk group had poor overall survival (OS) (Fig. 5C). ROC curves were generated to assess the predictive accuracy of the model for 20-year overall survival in breast cancer patients. In this prognostic model, the AUC value was 0.610 (Fig. 5D).

Relationship between CNV of immune genes and immune cell infiltration

**Fig. 6:**
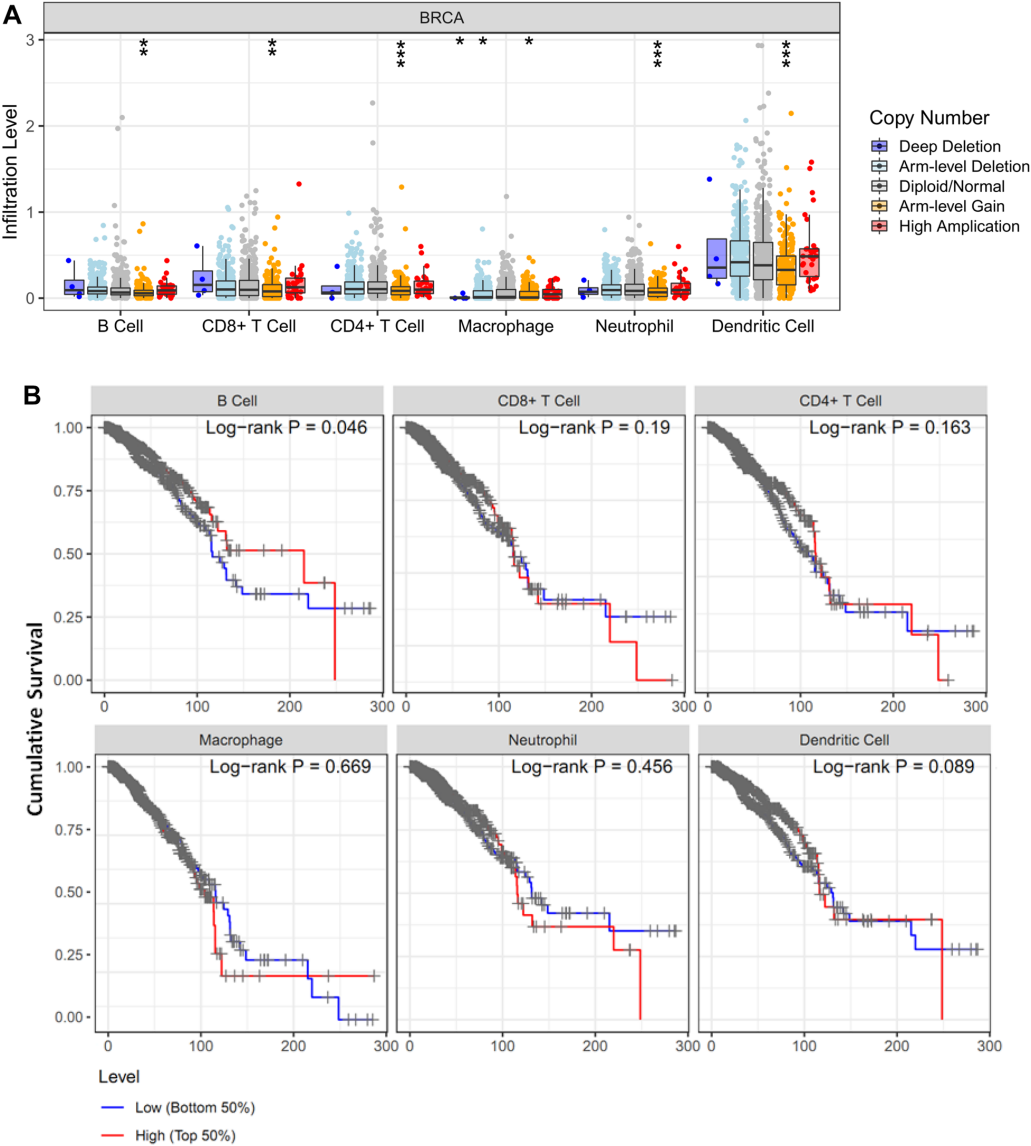
The correlation between CNV of immune genes and immune cell infiltration. **A** The arm-level gain probably inhibit the infiltration of immune cells. **B** Survival curves comparing the prognosis of patients with high and low of immune cells infiltration. The low B cell group corresponded to a poor survival prognosis in breast cancer patients. *, p < 0.05; **, *p* < 0.01; ***, *p* < 0.001

The TIMER database was used to investigate the correlation between CNVs of immune-related DEGs and immune cell infiltration in breast cancer patients. When *CCL18* varied in arm-level gain, the infiltration of B cells, CD8 + cells, CD4 + T cells, macrophages and neutrophils decreased significantly in breast cancer samples (Fig. 6A). The breast cancer patients in the low B cell group had a poor survival prognosis (Fig. 6B). *TRGC1* was not analyzed due to the limitation of data.

## Discussion

In this study, the landscape of different genetic mutations was explored in breast cancer. The results showed that TP53, PIK3CA, TTN and CDH1 were the most prominent mutated genes. TP53 plays a central role in human cancer pathogenesis and is hypermutated in almost all human cancers [18]. There is strong evidence that TP53 mutation is correlated with poorer overall and disease-free survival in breast cancer patients [19–21]. Recently, the comprehensive cancer genome analyses described that phosphoinositide-3 kinase (PI3K) pathway are frequently altered in human cancers [22, 23]. Mutations of PIK3CA lead to tumorigenesis and hyperactivity of the PI3K pathway [24]. TTN is another frequently mutated gene in a variety of human cancers, such as lung adenocarcinoma, lung squamous cell carcinoma and colon adenocarcinoma [25]. Double mutations of TTN and TP53 may induce breast cancer through regulating a common downstream pathway [26]. The CDH1 gene, located on chromosome 16q22.1, is a well-known tumor suppressor gene. The dysregulation, mutation or transcriptional silence of CDH1 gene probably cause breast cancer development [27]. These reports were consistent with our findings. We found that the low-TMB group had favorable overall survival (OS), being consistent with the results of a survival study in breast cancer conducted by Chen et al. [28]. TMB was significantly correlated with regional lymph node metastasis (N). However, there was no significant association between TMB and other clinical pathological characteristics in breast cancer.

A total of 598 DEGs were identified in breast cancer. KRT gene family members play an important role in breast cancer. We found that KRT83 and KRT1 were upregulated in the low-TMB group, while KRT4 was downregulated. Several studies have shown that the invasion and migration of cancer cells are related to abnormal expression of KRT genes [29–31]. We observed that DEGs were mainly involved in neuroactive ligand-interaction receptors according to KEGG enrichment analysis. The DEGs associated with the low-TMB group were mainly involved in the MAPK, mTOR, GnRH, TGF-beta and Hedgehog signaling pathways. The MAPK pathway is overactivated in various tumors. Many proteins that involve in MAPK pathway have been identified as oncogenic proteins [32]. Triple-negative breast cancer patients with MAPK/ERK signaling pathway upregulation have a poor survival prognosis [33]. The hedgehog pathway is associated with hormone receptor (HR +)-positive and triple-negative breast cancer patients [34]. Aberrant regulation of other signaling pathways, including mTOR, GnRH, and TGF-bate, could result in breast cancer development [35].

The lower expression of CCL18 and TRGC1 was significantly correlated with favorable survival outcomes in breast cancer, while their higher expression was associated with poor prognosis. CCL18 is produced abundantly by breast tumor-associated macrophages (TAMs), and its expression is also associated with tumor metastasis [36]. CCL18 promotes breast cancer cell invasion and metastasis through [activating/increasing?] Annexin A2 [37]. When CCL18 varied in arm-level gain, the infiltration levels of B cells, CD8 + cells, CD4 + T cells, macrophages and neutrophils decreased significantly in breast cancer samples. Breast cancer patients with low levels of B cells infiltration had a poor survival prognosis. The role of B cells in cancer is controversial in terms of immune cell infiltration. They have been reported to play both positive and negative roles in tumor immunity [38]. Recent years, B cells are considered to be a novel biomarker for ICB therapy and have been demonstrated to be important in ICB-driven antitumor responses because of their secretion of antibodies and helping T cell responses [39]. Daniel P et al. reported that the suppression effect of B cell on T cells may be due to the depletion of T cell subsets caused by antigen presentation, which in turn reduce the efficacy of ICB. The antibodies produced by B cells also play a key role in the ICI response [39]. IgG-secreted B cells can induce cytotoxicity through multiple mechanisms [40]. Daniel P et al. also observed that the efficacy of ICI disappeared when using mice with deficient Ig secretion [39]. These studies indicated that B cells mediate antitumor responses by activating T cells and producing antibodies.

## Conclusion

This study revealed that TMB could be a biomarker for predicting overall survival in immunotherapy in breast cancer patients. It was also correlated with immune cell infiltration. Of note, the high-TMB scores were correlated with a reduced survival rate in breast cancer patients. *CCL18* is an important gene in breast cancer, and the CNV of *CCL18* may reduce immune cell infiltration. Moreover, low B cell infiltration is correlated with poor survival prognosis in breast cancer patients.

## Data Availability

All data generated or analyzed during this study are included in this published article.
